# Where is My Spleen? – A Case of Splenosis Diagnosed Years Later after Splenectomy

**DOI:** 10.7759/cureus.2618

**Published:** 2018-05-13

**Authors:** Patricia Guzman Rojas, Jignesh Parikh, Anton Mahne, Priya Vishnubhotla, Juan J Oharriz

**Affiliations:** 1 Internal Medicine, University of Central Florida College of Medicine, Orlando, USA; 2 Pathology, Orlando VA Medical Center; 3 Radiology, Orlando VA Medical Center; 4 Medicine, Hematology-Oncology, Orlando VA Medical Center; 5 Gastroenterology, Orlando VA Medical Center

**Keywords:** splenosis, liver tumors

## Abstract

Hepatic splenosis was first described in 1939 and is a rare condition that results from splenic trauma or splenectomy.

A 43-year-old man with a past medical history significant for a prior splenectomy was admitted to the hospital due to right upper quadrant pain for two days. Magnetic resonance imaging (MRI) of the abdomen suggested features of hepatic adenoma, however, a percutaneous biopsy showed the mass within the liver to be a discrete collection of splenic tissue, apparently the result of a traumatic splenic rupture years ago.

Hepatic splenosis is a rare entity, and due to the asymptomatic nature of this condition, most cases are found incidentally after different imaging modalities are done. The management of this entity is based on conservative measures. We report this case to emphasize that in the appropriate clinical setting, hepatic splenosis should be considered in the differential diagnosis of a patient with a homogenous well-circumscribed liver mass.

## Introduction

Abdominal splenosis is the direct seeding or hematogenous spread of splenic tissue within the abdominal cavity. Hepatic splenosis was first described in 1939 by Buchbinder and Lipkoff [1]. It is a rare condition that results from splenic trauma or splenectomy.

## Case presentation

**Figure 1:**
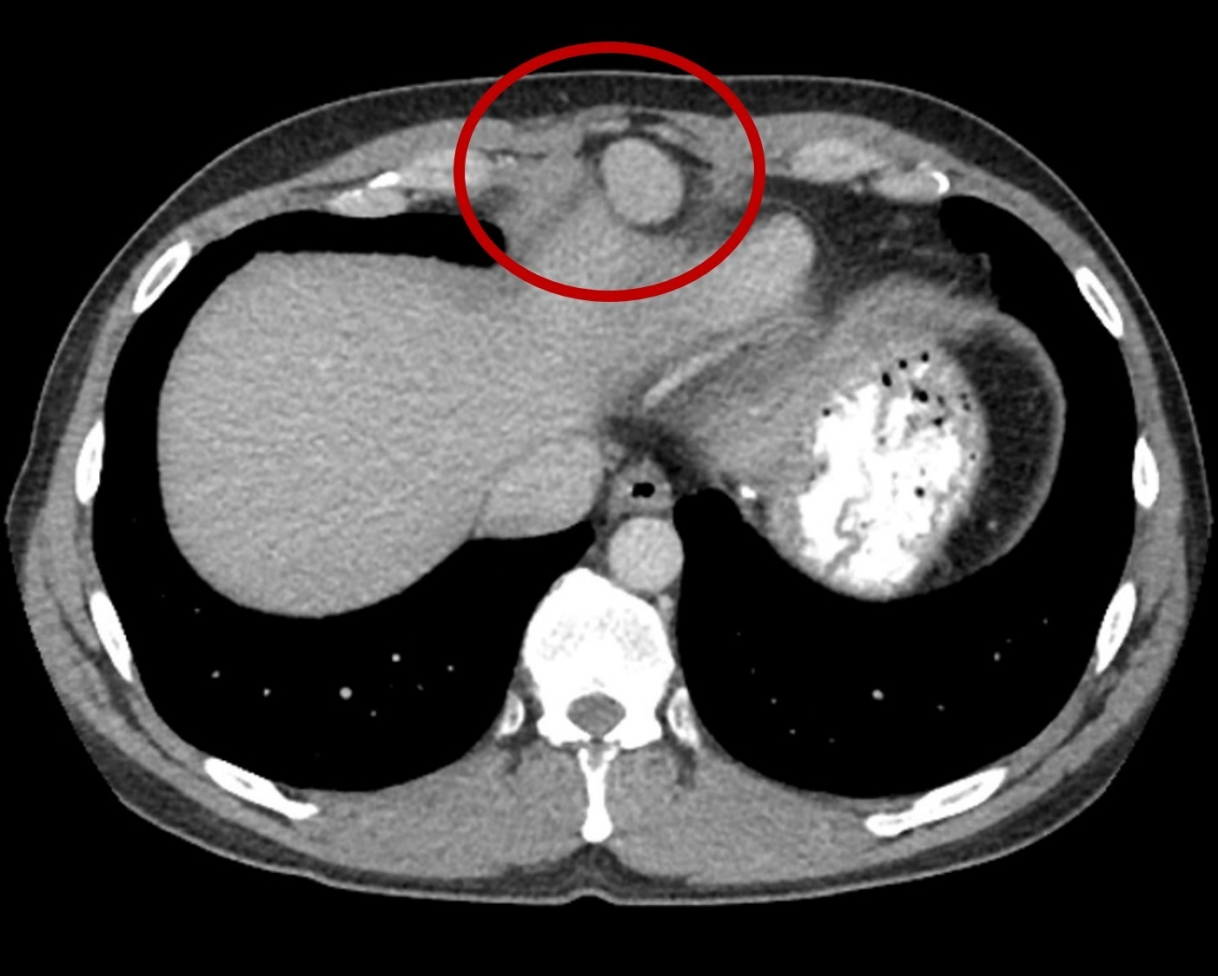
Computed tomography (CT) abdomen showing a solitary circumscribed mass (red circle) in segment 2 of the liver, measuring 2.5 x 2.0 cm and arising exophytically and anteriorly.

**Figure 2:**
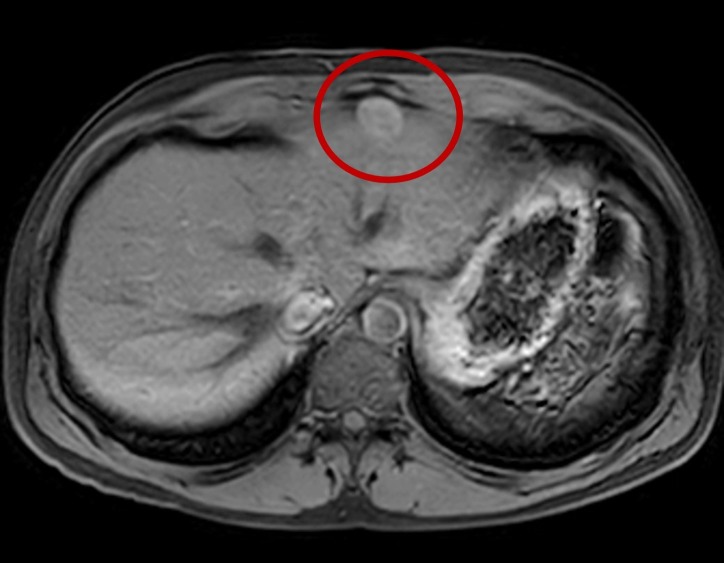
Magnetic resonance imaging (MRI) abdomen showing a mass (red circle) in segment 2 of the liver measuring 2.4 x 2.0 cm arising exophytically and anteriorly, similar to that seen in the previous computed tomography (CT) abdomen.

**Figure 3:**
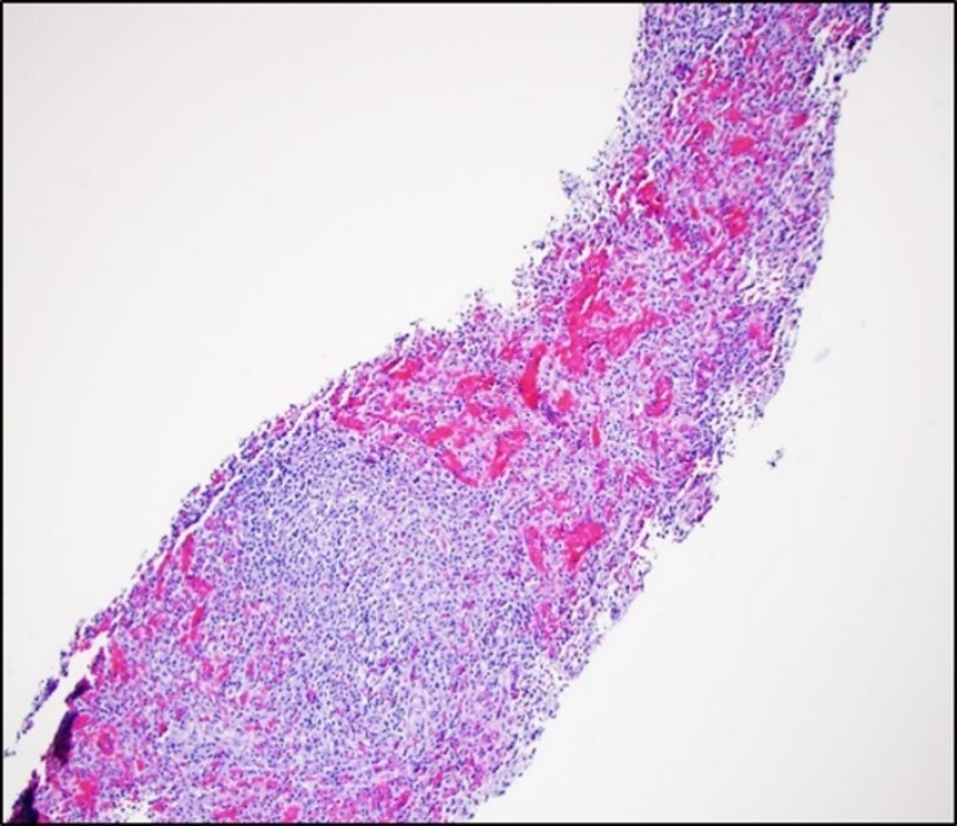
Hematoxylin and eosin stained liver biopsy sections revealing dilated capillaries and lymphoid aggregates that resemble splenic red and while pulp areas, respectively.

**Figure 4:**
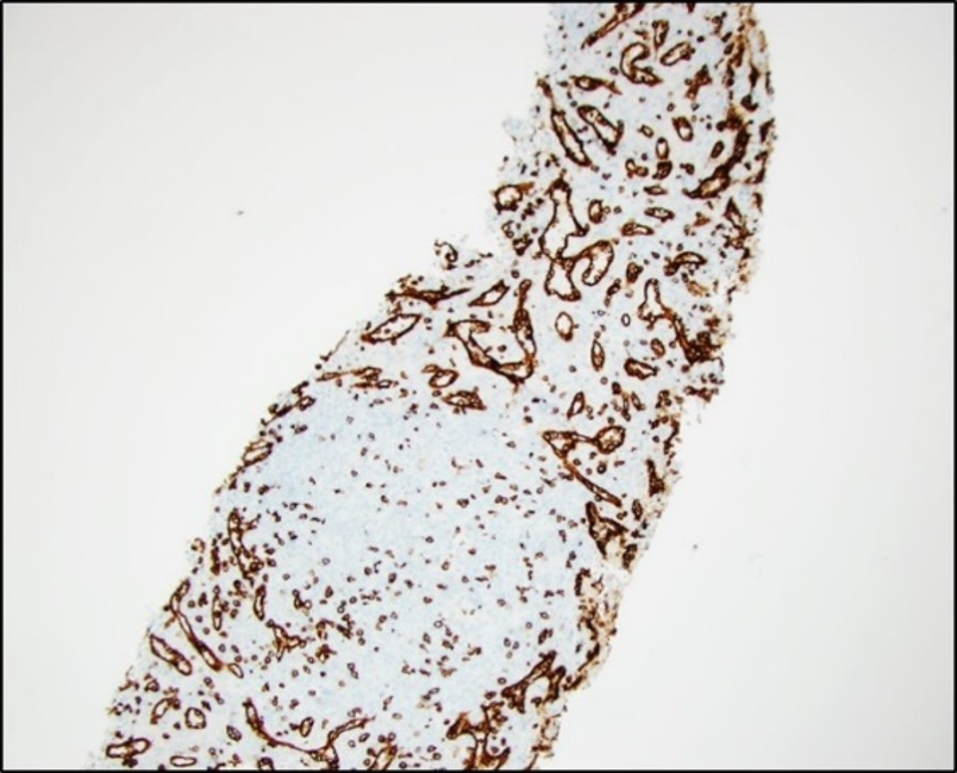
Liver biopsy showing CD8 stained liver biopsy sections revealing a red pulp architecture that resembles splenic red pulp areas.

**Figure 5:**
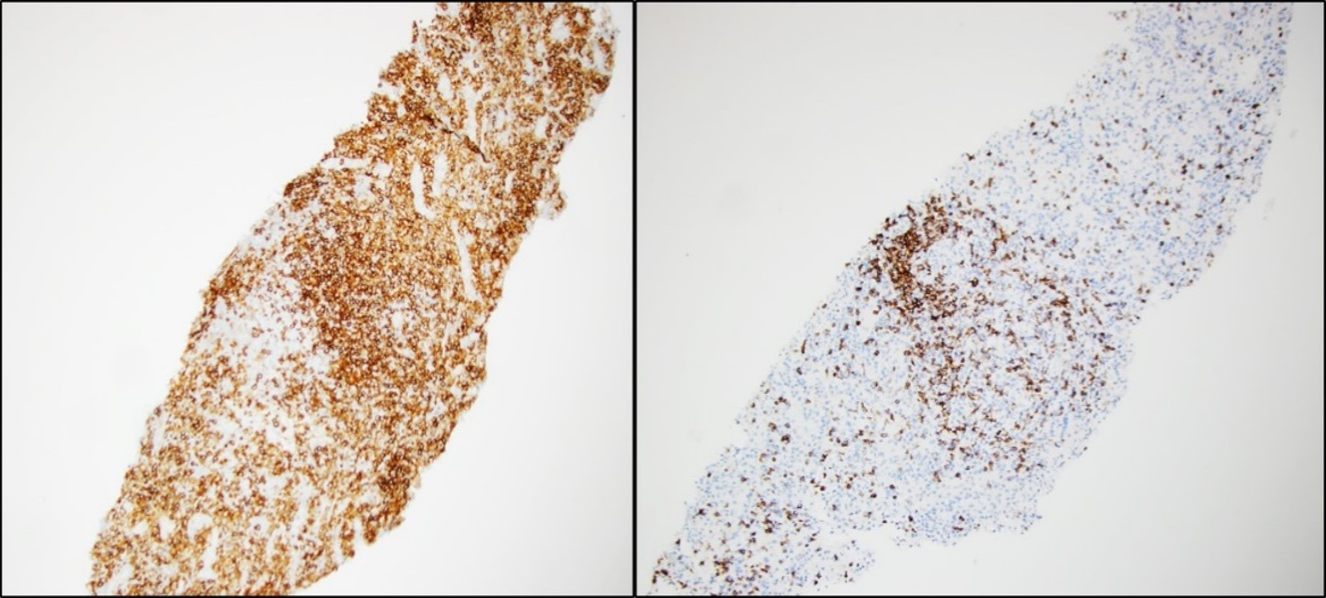
CD20 stained (Left panel) and CD3 stained (Right panel) liver biopsy sections showing B-cell aggregates and T-lymphocytes, respectively.

We present a 43-year-old man with a past medical history only significant for a prior splenectomy who was admitted to the hospital due to right upper quadrant pain for two days. This was described as a dull “liver pain” in the right upper quadrant area. On physical exam, there was evidence of a midline scar, the abdomen was soft with mild tenderness to palpation of the right upper quadrant and the liver span was approximately 10 cm in the mid-clavicular line by percussion. A complete blood count and a basic metabolic panel were normal; however, alanine transaminase (ALT) and aspartate transaminase (AST) showed a mild elevation of 66 U/L and 51 U/L, respectively. Serum bilirubin levels and alkaline phosphatase levels were within normal limits. Due to the reported complaint of right upper quadrant pain and the associated abnormal liver function tests, an abdominal ultrasound (US) was ordered. This showed fatty liver disease and a left liver lobe isoechoic liver mass. A computed tomography (CT) triple phase abdomen scan was done demonstrating a 2.5 cm exophytic mass in the liver in segment 2 (Figure 1). The next day of admission, the patient’s pain improved with analgesia. As no clear diagnosis was made, he was later discharged with an intention to perform an elective abdominal magnetic resonance imaging (MRI). This MRI revealed a single mass in segment 2 of the liver, with features of a hepatic adenoma (Figure 2). The surgical team was consulted and evaluated the patient and an elective percutaneous liver biopsy was performed. Examination of hematoxylin and eosin (H&E) stained sections revealed histological evidence of splenic tissue with distinct red and white pulp areas, with evidence of passive congestion (Figure 3). The red pulp included thin-walled venous sinusoids that were congested with red blood cells that were positive for CD8 stains (Figure 4), with surrounding macrophages and few lymphocytes. The white pulp included thickened meshwork of cords showing arterioles sheathed by predominantly small T lymphocytes (CD3+) and scattered B-cell aggregates (CD20+), consistent with splenic Malpighian corpuscles (Figure 5). On further questioning, the patient reported he had an exploratory laparotomy with subsequent emergent splenectomy at the age of 16 years due to a motor vehicle accident which caused a splenic rupture. As the patient was diagnosed with hepatic splenosis and was at this point asymptomatic, his benign diagnosis was explained, and no further workup was needed.

## Discussion

Splenosis has been widely reported around the world with an incidence of 16-67% after traumatic splenic rupture or splenectomy. The etiology of splenosis begins after an initial splenic rupture or splenectomy which causes splenic pulp to scatter into the peritoneal cavity via direct spread. The period from a splenectomy or splenic trauma to the diagnosis of splenosis varies from months to years. Mescoli et al. reported two cases and reviewed 26 previously described cases of liver splenosis [2], and found an average time between the traumatic event to diagnosis of 29 years.

In general, the most common locations of splenosis are the serosal surfaces of the small intestine, greater omentum, parietal peritoneum, large intestine, mesentery and under the surface of the diaphragm [3]. The number of splenosis nodules varies in quantity, from a couple to up to 400 nodules and the size of these nodules varies from a few millimeters to 12 cm [4]. However, nodules rarely become larger than 3 cm due to the constraints of their local blood supply which limit their growth [3].

Another entity that arises from ectopic splenic tissue is the accessory spleen. Accessory spleen is a congenital anomaly where splenic nodules are found separated from the main body of the spleen, with normal splenic tissue histology. These are supplied by the splenic artery and can be found close to the splenopancreatic or gastrosplenic ligament [5].

Hepatic splenosis, such as that seen in our patient, is a discrete collection of splenic tissue confined to the liver. It is a rare entity with an unknown incidence and prevalence. There are several postulated mechanisms for the development of hepatic splenosis. One such mechanism includes the dispersion of splenic tissue (either after splenic trauma or splenectomy) into the peritoneum which eventually causes direct seeding of splenic pulp into the liver. Another mechanism includes splenic derived erythrocytic progenitor cells embolizing into the liver via the portal vein. Due to the relative hypoxia of the erythrocytic progenitor cells in the liver, there is a reflex erythropoiesis of these cells which further stimulates their growth [5,6].

In the general population, the differential diagnosis of hepatic splenosis includes hepatic adenoma, hemangioma, focal nodular hyperplasia, peritoneal mesothelioma, abdominal lymphoma, hepatocellular carcinoma, liver metastasis and endometriosis [2,7].

Hepatic splenosis shows a clinical picture that is frequently asymptomatic. However, it can cause associated manifestations like nonspecific abdominal pain, an enlarged abdominal mass, intestinal obstruction or even gastrointestinal hemorrhage [5].

Currently there are several imaging methods available to identify liver lesions which are suggestive of hepatic splenosis. Abdominal US shows hepatic splenosis nodules to be hypoechoic, homogenous, solid, and/or well-circumscribed masses [8]. On the other hand, CT imaging and MRI are able to localize the lesions, but not the etiology of these, with MRI having a better sensitivity for diagnosis [5,9]. Non-contrast CT imaging shows a hypodense mass that becomes hyperdense in the arterial phase after contrast administration. MRI without contrast shows a homogeneously hypointense mass in T1-weighted images, which turn hyperintense after contrast administration. Additionally, on MRI there is a rim surrounding the mass, which is a suggestive finding of splenosis. MRI with intravenous administration of superparamagnetic iron oxide (SPIO) is a technique used to help differentiate splenosis from hepatic malignancies. This is based on the ability of the reticuloendothelial system to take up SPIO [5]. Finally, scintigraphy with technetium-99m-labeled red blood cells is the most sensitive diagnostic modality to diagnosis hepatic splenosis, with a reported sensitivity of up to 58-66% [7,10].

Despite the multiple imaging modalities available to assist in the diagnosis of hepatic splenosis, the gold standard for diagnosis is histological confirmation. Different immunohistochemical stainings are used to identify splenic tissue and include CD20 staining which is used to identify B lymphocytes, CD3 stains which identify T lymphocytes that are formed around the periarteriolar sheath and the CD8 stain which highlights sinus endothelial cells.

The management of this entity is based on conservative measures, and surgery is indicated only when the patient is symptomatic [6]. Our patient’s abdominal pain was self-limited and for this reason, no treatment was needed.

## Conclusions

We report this case to highlight the clinical features of hepatic splenosis. In the appropriate clinical setting, hepatic splenosis should be considered in the differential diagnosis of a patient who presents with a homogenous well-circumscribed liver mass.
